# The Impact of Dietary Habits and Maternal Body Composition on Human Milk Microbiota—Polish Pilot Study

**DOI:** 10.3390/molecules30132723

**Published:** 2025-06-25

**Authors:** Agnieszka Bzikowska-Jura, Anna Koryszewska-Bagińska, Małgorzata Konieczna, Jan Gawor, Robert Gromadka, Aleksandra Wesołowska, Gabriela Olędzka

**Affiliations:** 1 Laboratory of Human Milk and Lactation Research at Human Milk in the Specialistic Holy Family Hospital in Warsaw, Department of Medical Biology, Faculty of Health Sciences, Medical University of Warsaw, 00-575 Warsaw, Poland; aleksandra.wesolowska@wum.edu.pl; 2 Department of Medical Biology, Faculty of Health Sciences, Medical University of Warsaw, 00-575 Warsaw, Poland; anna.koryszewska-baginska@wum.edu.pl (A.K.-B.); malgorzata.konieczna@wum.edu.pl (M.K.); gabriela.oledzka@wum.edu.pl (G.O.); 3 Institute of Biochemistry and Biophysics, Polish Academy of Sciences (IBB PAS), Pawińskiego 5a, 02-106 Warsaw, Poland; gaworj@ibb.waw.pl (J.G.); robert@ibb.waw.pl (R.G.)

**Keywords:** human milk, breastfeeding, human milk microbiota, maternal diet, infants’ feeding, Firmicutes, Bacteroidetes

## Abstract

Human milk (HM) is a complex biological fluid that plays a significant role in infant health, influenced by maternal dietary habits and body composition. This study aimed to explore how maternal diet and nutritional status affect the microbial composition of HM. In this pilot study, 15 mothers were recruited from a maternity ward and assessed for dietary habits through a semi-structured food frequency questionnaire and a 3-day dietary record. Maternal body composition was evaluated using bioelectrical impedance analysis. HM samples were collected for microbiota analysis, focusing on the diversity and composition of bacterial communities via 16S rRNA sequencing. The study identified that maternal nutrient intake significantly correlated with the composition of HM microbiota. Specifically, Firmicutes abundance showed positive correlations with animal protein (τ = 0.39; *p* = 0.043), total carbohydrates (τ = 0.39; *p* = 0.043), and vitamin A (τ = 0.429; *p* = 0.026). Bacteroidota was positively correlated with retinol (τ = 0.39; *p* = 0.043). Higher consumption of dietary fiber (>24 g/day) did not yield significant differences in bacterial composition compared to lower intake (<24 g/day) (*p* = 0.8977). Additionally, no significant differences were found in overall bacterial abundance across different maternal characteristics such as age, mode of delivery, or breastfeeding type. This study underscores the importance of maternal diet in shaping the HM microbiota, which may have implications for infant health. Dietary modifications during lactation could be a strategic approach to promote beneficial microbial colonization in HM. Further research is warranted to confirm these findings and explore the underlying mechanisms.

## 1. Introduction

Human milk (HM) is a biologically complex fluid with diverse components and is crucial in shaping infant health. Beyond providing essential macro- and micronutrients, it contains active biological components and a diverse microbiota that significantly influences the development of the neonatal gut microbiome and immune system [1,2,3]. Recent research has highlighted the significance of HM microbiota in early-life colonization [4,5,6], with evidence suggesting that maternal factors such as diet, health status, mode of delivery, and antibiotic use impact microbial composition [7,8,9]. Understanding these factors is essential for optimizing maternal health and developing targeted nutritional strategies. The HM microbiota is composed of various bacterial genera, including *Staphylococcus*, *Streptococcus*, *Bifidobacterium*, and *Lactobacillus*, which contribute to establishing a balanced infant gut microbiome [10]. These microbes are thought to originate from multiple sources, including the maternal gut via the entero-mammary pathway [11], retrograde transfer from the infant’s oral cavity during breastfeeding [12], and environmental exposure [9,13]. Studies have shown that the HM microbiota plays a role in immune modulation, helping to establish a balanced immune response and reducing the risk of allergies, infections, and autoimmune disorders [14].

Nutritional components of HM, such as human milk oligosaccharides (HMOs), lactoferrin, and polyunsaturated fatty acids, play a key role in modulating microbial diversity and function [15]. HMOs, for instance, serve as prebiotics that selectively promote the growth of beneficial bacteria, particularly *Bifidobacterium* species, which dominate the gut microbiota of breastfed infants [16]. Beyond supporting beneficial bacteria, HMOs also act as decoy receptors for pathogens, preventing their attachment to the infant’s gut lining [15].

Apart from oligosaccharides, maternal diet is increasingly recognized as a key determinant of HM microbiota composition. Studies suggest that diets rich in fiber, fermented foods, and polyphenols enhance microbial diversity and beneficial taxa [17,18], while high-fat and Western-style diets may negatively affect microbial composition and functionality [19]. Furthermore, probiotic supplementation during pregnancy and lactation has been proposed as a strategy to modify HM microbiota, with potential benefits for both mother and infant [20].

Given the growing interest in the role of HM microbiota in infant development, further research is needed to elucidate the specific factors influencing its composition and functional implications. Understanding how maternal diet, genetics, and lifestyle impact milk microbiota could help refine dietary recommendations and interventions that support optimal microbial colonization in infants. In this context, we present a pilot study aimed at exploring the relationship between maternal dietary habits, nutritional status, and the microbiota of HM, providing foundational data for future, larger-scale research.

## 2. Results

### 2.1. The Characteristics of the Study Group

Maternal–infant characteristics are presented in Table 1. The mean maternal age was 32.1 ± 5.0 years, and almost all the women had a normal body weight before pregnancy (*n* = 14, 93%) and during the first month postpartum (*n* = 13, 87%). In the study group, 53.3% of deliveries were by cesarean section, and 67% of mothers (*n* = 10) gave birth to a baby girl. Considering body composition analysis, most of the women (*n* = 9, 60%) had a lower than recommended content of total body water (below 50% of total body weight). Fat mass assessment could not to be evaluated due to the lack of recommendations for postpartum women in confinement, as higher fat distribution during this period differs significantly from pre-pregnancy norms. Therefore, reference values for healthy, non-pregnant adult women should not be applied in the first weeks postpartum.

Maternal dietary intake of selected nutrients based on 3-day dietary records is presented in Table 2. The average total protein intake was 81.91 g (SD ± 24.2), with 17.06% of energy derived from protein. Total fat intake averaged 68.7 g (SD ± 17.91), contributing 31.54% of energy, while total carbohydrates were 260.5 g (SD ± 66.3), providing 51.09% of energy. Among micronutrients, sodium was 2862 mg (SD ± 1043), potassium was 3202 mg (SD ± 893.5), and calcium was 818 mg (SD ± 354.3). The average intake of vitamin D was 4.266 μg (SD ± 3.71), and vitamin C was 137.3 mg (SD ± 100.9).

### 2.2. Microbial Diversity in Human Milk Samples

**Figure 1:**
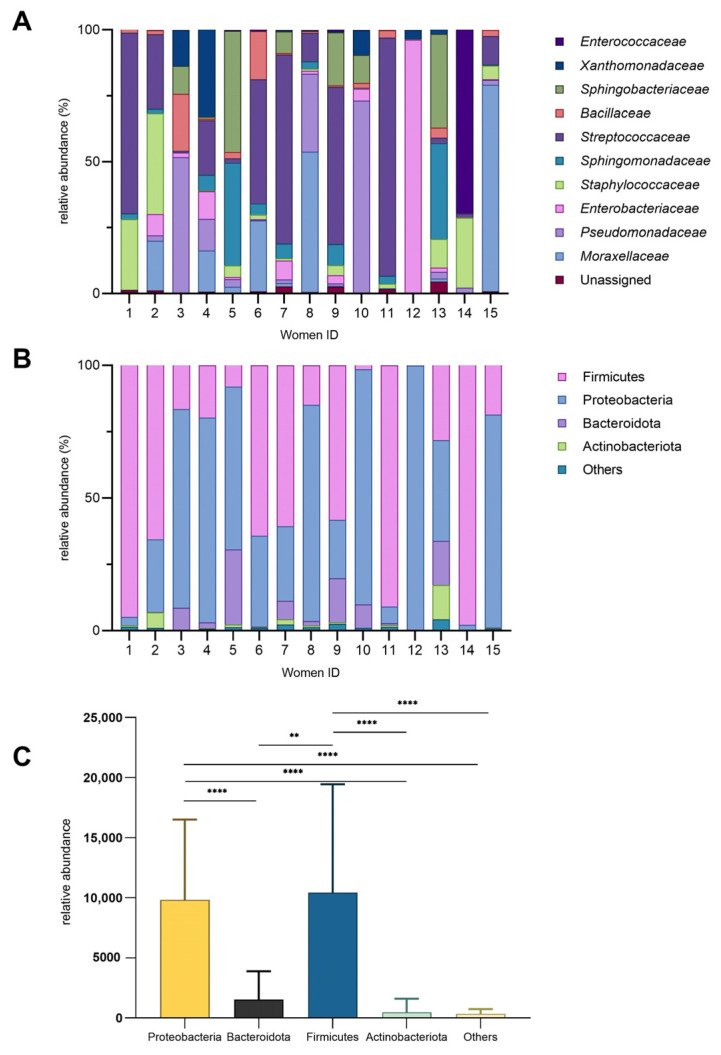
Bacterial diversity in human milk. (**A**) Relative abundance (%) of bacterial families across individual donors. (**B**) Relative abundance of major phyla (%). (**C**) Phylum-level diversity profile (mean ± SD; ** *p* = 0.002; **** *p* < 0.0001).

The microbial composition of HM samples collected from 15 women was characterized, and the inter-individual variability was observed (Figure 1). For instance, the bacterial families (Figure 1A) that accounted for over 50% of the total bacteria in certain milk samples were *Streptococcaceae*, *Enterobacteriaceae*, *Moraxellaceae*, *Enterococcaceae*, and *Pseudomonadaceae*. *Enterococcaceae* and *Enterobacteriaceae* exhibited higher relative abundances (69% and 96%, respectively) in just one milk sample (14 and 12), while they were present at lower levels or nearly absent levels (below 10%) in the remaining samples. Notably, women 1, 11, 12, and 14 had the least microbial diveristy in milk samples. *Pseudomonadaceae* was particularly abundant in women 3, 8, and 10 (approximately 30–70%) but minimal in others. *Staphylococcaceae* showed moderate abundance in women 1, 2, and 14 (26–38%) yet was nearly absent in women 5 and 15. *Bacillaceae* was more prominent in women 3 and 6 (18–21%) but low in other samples. The bacterial family that presented the most in HM samples was *Streptococcaceae*. Samples 5 and 13 displayed similar microbial profiles, characterized by comparable levels of *Sphingomonadaceae* and *Sphingobacteriaceae*, with only slight differences in the abundance of *Staphylococcaceae* between them.

At the phylum level, Proteobacteria and Firmicutes dominated most samples, while Bacteroidota and Actinobacteriota were consistently less abundant (Figure 1B). The relative abundance of these phyla varied significantly among individuals, highlighting inter-individual differences in microbial composition. Notably, Firmicutes were significantly more abundant than Bacteroidota (Figure 1C) (*p* = 0.002). The “Others” category constituted a minor fraction, generally less than 5%, across all samples.

### 2.3. The Impact of Dietary Fiber Intake on Human Milk Microbiota

**Figure 2:**
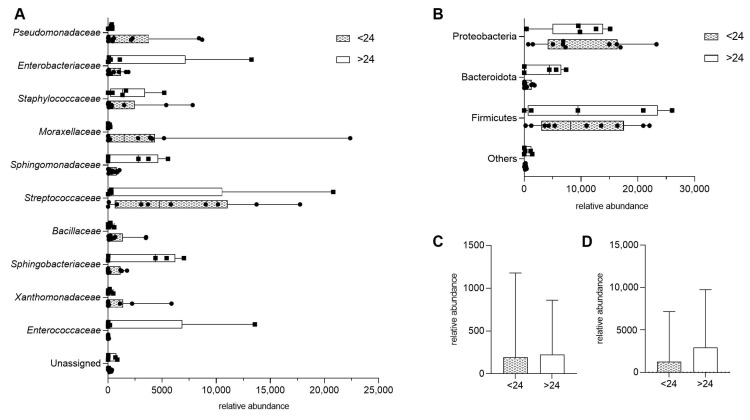
Impact of maternal fiber intake on human milk microbiota, presented as means with min and max. (**A**) Bacterial composition at the family level in samples from donors consuming less than 24 units of fiber per day (<24) and those consuming more (>24). (**B**) Bacterial composition grouped by phylum, comparing the same two fiber intake groups. (**C**,**D**) Total bacterial abundance by dietary fiber intake, presented as median with interquartile range; *p* > 0.05.

To assess whether dietary fiber intake influences microbial diversity in HM, we compared samples from donors consuming less than 24 g of fiber per day (<24) with those consuming more (>24). There were no statistically significant differences in bacterial composition at the family level (Figure 2A). Similarly, when bacterial taxa were grouped by phylum, no significant differences were observed between the two fiber intake groups (Figure 2B).

Further analysis examined the overall microbial diversity with fiber intake. No significant differences were found between the two fiber consumption groups when bacterial families (*p* = 0.8977) and bacterial phylum (*p* = 0.6857) were compared (Figure 2C,D).

### 2.4. Associations Between Selected Maternal Nutrient Intake and Microbial Composition

**Figure 3:**
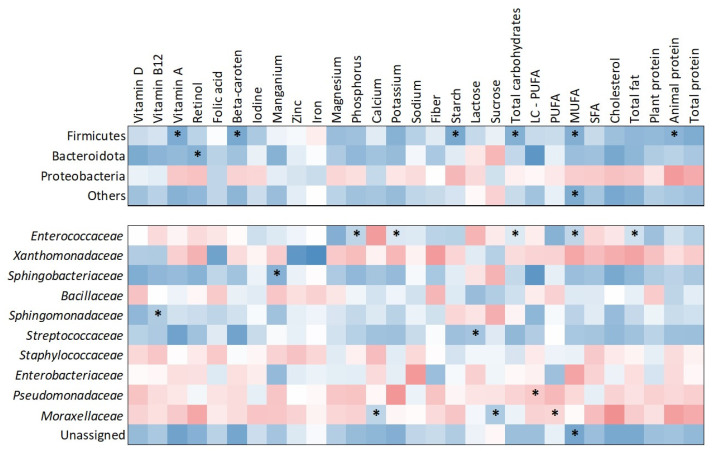
Heatmap of correlations between nutrient intake and milk microbiota composition at phylum and bacterial family levels. Warm colors indicate negative correlations, while cool colors indicate positive correlations. Significant correlations (*p* < 0.05) are marked with asterisks.

The heatmap (Figure 3) illustrates correlations between nutrient intake and bacterial abundance at both the phylum and family levels. At the phylum level, Firmicutes showed significant positive correlations with several dietary components, including starch (τ = 0.505; *p* = 0.009), vitamin A (τ = 0.429; *p* = 0.026), beta-carotene (τ = 0.429; *p* = 0.026), total carbohydrates (τ = 0.390; *p* = 0.043), monounsaturated fatty acids (MUFA) (τ = 0.429; *p* = 0.026), and animal protein (τ = 0.390; *p* = 0.043). Bacteroidota was positively correlated with retinol intake (τ = 0.390; *p* = 0.043). At the family level, *Enterococcaceae* showed significant positive correlations with phosphorus (τ = 0.431; *p* = 0.030), potassium (τ = 0.471; *p* = 0.018), total carbohydrates (τ = 0.390; *p* = 0.049), MUFA (τ = 0.451; *p* = 0.020), and total fat (τ = 0.390; *p* = 0.049). *Sphingobacteriaceae* was positively correlated with manganese intake (τ = 0.426; *p* = 0.036) and *Sphingomonadaceae* with vitamin B12 intake (τ = 0.448; *p* = 0.020). *Streptococcaceae* showed a significant positive association with lactose intake (τ = 0.448; *p* = 0.020). *Pseudomonadaceae* demonstrated a negative correlation with long-chain polyunsaturated fatty acids (LC-PUFA) (τ = –0.383; *p* = 0.048). Additionally, *Moraxellaceae* was positively associated with calcium (τ = 0.471; *p* = 0.015) and sucrose (τ = 0.375; *p* = 0.042) while showing a negative correlation with polyunsaturated fatty acids (PUFA) (τ = –0.413; *p* = 0.033).

**Figure 4:**
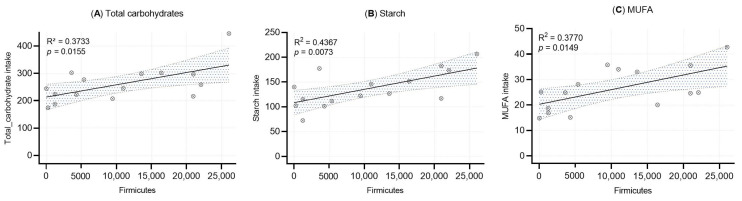
Simple linear regression analysis between Firmicutes abundance and (**A**) total carbohydrate intake, (**B**) starch intake, and (**C**) MUFA intake. Each dot represents an individual sample. Shaded areas represent the 95% confidence intervals, with R^2^ and *p*-value included.

Linear regression analyses were performed to evaluate the associations between the relative abundance of Firmicutes in human milk and the intake of total carbohydrates, starch, and monounsaturated fatty acids (MUFA) (Figure 4). Statistically significant positive correlations were observed for all three nutrients: total carbohydrates (R^2^ = 0.373, *p* = 0.0155), starch (R^2^ = 0.437, *p* = 0.0073), and MUFA (R^2^ = 0.377, *p* = 0.0149).

### 2.5. Associations Between Food Frequency Intake and Bacterial Taxa

The results presented in Table 3 summarize the associations between significant bacterial taxa in HM and higher dietary intake of specific food groups. The analysis highlights several statistically significant relationships, with *Sphingomonadaceae* (*p* = 0.005) associated with animal milk consumption and *Staphylococcaceae* (*p* = 0.002) linked to plant oil intake. Furthermore, *Bacillaceae* appears in multiple food groups, including oily fishes (*p* = 0.069), lean fishes (*p* = 0.052), and butter (*p* = 0.103), suggesting a potential dietary influence on its presence in HM microbiota. Similarly, *Sphingobacteriaceae* shows associations with lean fish (*p* = 0.131), buckwheat (*p* = 0.035), and peanuts (*p* = 0.029). Wholegrain consumption was notably linked to three bacterial families: *Sphingobacteriaceae* (*p* = 0.111), *Xanthomonadaceae* (*p* = 0.059), and *Enterobacteriaceae* (*p* = 0.018).

### 2.6. Relationships Between Maternal Factors and Microbial Composition

**Figure 5:**
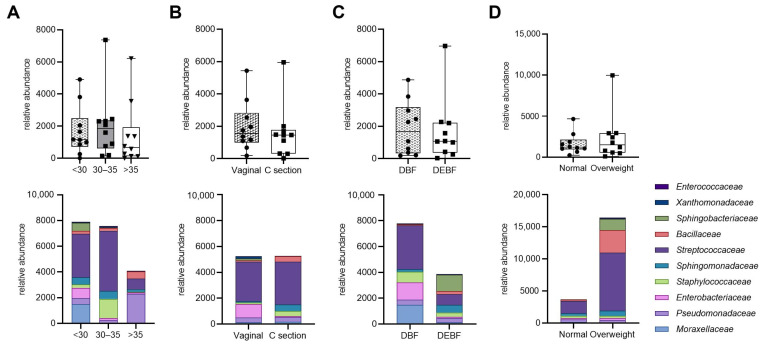
Relative abundance of bacterial families in human milk samples based on maternal characteristics. The top row shows bacterial abundance comparisons across different maternal factors: (**A**) age; (**B**) type of delivery; (**C**) breastfeeding method; (**D**) BMI (median and interquartile ranges. The bottom row displays the corresponding bacterial composition at the family level (medians). DBF—direct breastfeeding; DEBF—direct breastfeeding and feeding with expressed milk.

Figure 5 illustrates the relative abundance of bacterial families in HM samples based on maternal characteristics and breastfeeding method. Although differences in bacterial abundance across various factors such as age, type of delivery, breastfeeding method, and BMI were observed, they were not statistically significant. Notably, milk from mothers under 35 years tended to have higher bacterial abundance compared to those over 35. Additionally, vaginal delivery was associated with increased levels of *Enterobacteriaceae*, while direct breastfeeding showed a higher median bacterial load than mixed feeding methods. Overweight mothers also exhibited a trend towards greater abundance of *Bacillaceae* and *Streptococcaceae* compared to mothers with normal weight. These findings suggest that while maternal factors may influence the composition of human milk microbiota, the observed differences require further investigation due to their lack of statistical significance.

**Figure 6:**
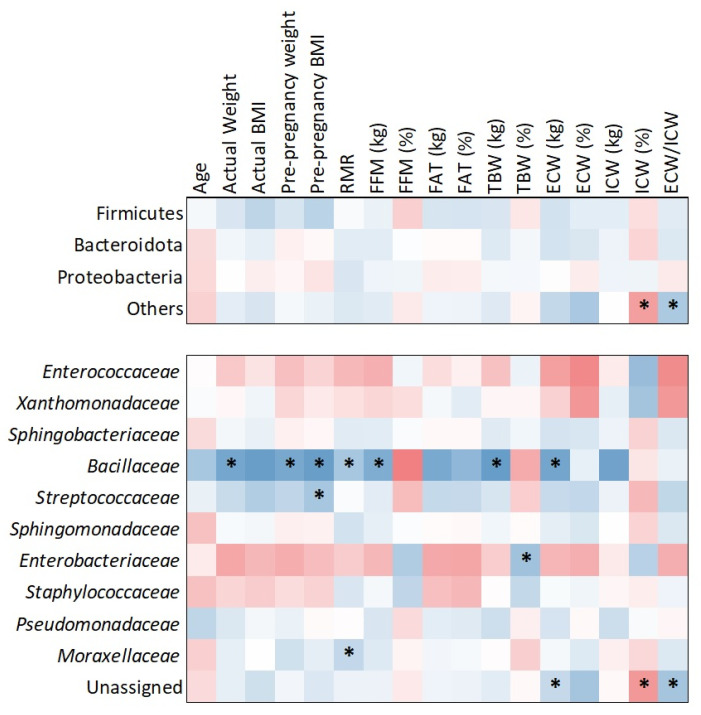
Correlations between bacterial families and maternal factors. Significant correlations (*p* < 0.05) are marked with asterisks. Cold colors indicate positive correlations and warm negative.

The heatmap analysis (Figure 6), based on Kendall tau correlation, revealed associations between bacterial families and maternal body composition parameters. Notably, *Bacillaceae* demonstrated positive and statistically significant correlations with several maternal metrics, including actual weight (τ = 0.468; *p* = 0.017), pre-pregnancy weight (τ = 0.537; *p* = 0.006), pre-pregnancy BMI (τ = 0.440; *p* = 0.023), resting metabolic rate (RMR) (τ = 0.529; *p* = 0.006), fat-free mass (FFM) (τ = 0.517; *p* = 0.007), total body water (TBW) (τ = 0.498; *p* = 0.010), and extracellular water (ECW) (τ = 0.574; *p* = 0.003).

In addition to *Bacillaceae*, other significant associations were observed. *Streptococcaceae* was positively correlated with pre-pregnancy BMI (τ = 0.390; *p* = 0.043), *Moraxellaceae* with RMR (τ = 0.386; *p* = 0.047), and *Enterobacteriaceae* with TBW (τ = 0.425; *p* = 0.029). While *Bacillaceae* exhibited the strongest and most consistent correlations, these additional findings suggest broader microbiota associations with maternal body composition. No significant phylum-level correlations were detected in relation to maternal anthropometric or metabolic factors.

## 3. Discussion

HM remains the subject of ongoing scientific research. Experts are discovering more about its composition and, above all, its health-promoting properties. Numerous studies have shown that, in addition to key nutrients and bioactive substances, HM also contains a diverse range of microorganisms, including bacteria, viruses, fungi, and archaea [21,22].

A non-culture method was used to determine the presence of bacteria in HM samples from 15 Polish women to their body composition and dietary habits. We identified specific relationships between maternal nutrient intake, weight, body composition, and the microbial profile of HM. In contrast, other factors, including maternal age, mode of delivery, and breastfeeding type, did not show a measurable effect. These findings highlight the potential for dietary modifications during lactation to shape the HM microbiota beneficially.

In agreement with previous data from other populations [9,23,24], we found that Firmicutes and Proteobacteria, followed by Bacteroidota and Actinobacteriota, were the most abundant phyla. Even though only mature milk was examined in our study, and all samples were collected from the same geographic region, notable differences in the relative abundance of these phyla were observed. Such interindividual variability has already been reported and, although little studied, has been related to a number of factors such as geographical location, lactation stage, and delivery method [13,25,26,27]. Notably, we identified a statistically significant Firmicutes dominance over Bacteroidota, consistent with previous reports identifying Firmicutes as the dominant phylum in HM. This may reflect selective ecological pressures unique to the mammary gland environment. The Firmicutes to Bacteroidetes ratio has previously been recognized as an important marker of gut microbiome composition [28]. The low relative abundance of Bacteroidota in HM, characteristically present in the gut microbiome, may indicate a niche-specific adaptation to the unique immunological and biochemical nature of HM. This interpretation can be supported by studies showing that approximately 25–30% of the infants’ gut microbiota originates from HM [6].

Also at the family level, the HM microbiome revealed significant individual donor variation. The composition was highly diverse, with different patterns of dominance between samples. The ten most abundant bacterial families detected in our study were *Streptococcaceae*, *Pseudomonadaceae*, *Moraxellaceae*, *Enterobacteriaceae*, *Sphingobacteriaceae*, *Sphingomonadaceae*, *Staphylococcaceae*, *Xanthomonadaceae*, *Bacillaceae*, and *Enterococcaceae*. Notably, some families exhibited extreme sample-specific dominance (e.g., *Enterobacteriaceae*), while others like *Streptococcaceae* and *Pseudomonadaceae* were consistently present across multiple samples. These results are generally consistent with those of previous studies [7,9,24,26,29,30,31]. Furthermore, as noted by Bode et al. [32], HM microbiota exhibits a high degree of interindividual variability, with only a limited subset of taxa shared across all participants.

In our study, the mode of delivery did not correlate with HM microbiota composition. Although numerous studies [26,33,34,35,36] have investigated whether the HM microbiome differs between vaginal and caesarean deliveries, the question remains unresolved. Findings from Canada [26] and China [36] are consistent with our results, showing no significant impact of delivery mode on the HM microbiota. However, one study [34] indicated that HM samples from mothers who underwent nonelective caesarean sections were more similar to those from vaginal deliveries than to samples obtained from elective caesarean sections. This suggests that physiological processes during childbirth may play a crucial role in shaping the HM microbiome. In contrast, an Italian study [37] based on the analysis of colostrum samples (days 0–3) revealed that vaginal deliveries were associated with higher levels of *Streptococcus* and lower levels of *Staphylococcus* and *Pseudomonas* compared to caesarean sections. In addition to the variations in the abundance of specific bacterial genera, interactions among microorganisms, as outlined by the mathematical model, showed significant differences between colostrum from caesarean and vaginal deliveries. These findings are fascinating, as they may suggest that bacterial interactions in HM may vary according to the delivery method. Such interactions could potentially influence the pathogenicity or beneficial roles of specific bacteria present.

Considering that individuals with obesity typically exhibit a less diverse gastrointestinal microbiome compared to those with a healthy weight [38], the HM microbiome of lactating women may also be influenced by obesity [36]. Cabrera-Rubio et al. [34] found that a higher maternal BMI was associated with increased levels of *Lactobacillus* in colostrum (r = 0.600, *p* = 0.026). Additionally, higher levels of Staphylococcus (r = 0.560, *p* = 0.038) and lower levels of *Bifidobacterium* in mature HM (six months postpartum) (r = −0.651, *p* = 0.012) were also linked to a higher maternal BMI. Similar results were reported by Lundgren et al. [39], who observed that higher maternal pre-pregnancy BMI was related to increased odds of belonging to BMT1 (characterized by a high abundance of *Streptococcus* and *Staphylococcus* genera) compared to BMT2 (where *Streptococcus* predominant taxa). However, the samples exhibited greater overall diversity compared to those in BMT1. In our study, due to the numerous limitations associated with BMI and its potential failure to accurately reflect the true nutritional status of women, we conducted a body composition analysis. We observed that at the family level, *Bacillaceae* showed several positive correlations with maternal body composition parameters (e.g., fat-free mass and total body water) (Figure 6). Moreover, the association with obesity is further supported by the observation that overweight women exhibited a higher abundance of *Bacillaceae* compared to women of normal weight (Figure 5D). This finding aligns with the results of a detailed correlation analysis, which demonstrated a positive relationship between BMI and *Bacillaceae* abundance (Figure 6). Furthermore, mothers with a BMI greater than 25.0 kg/m^2^ exhibited a trend toward increased abundance not only of *Bacillaceae* but also of *Streptococcaceae* and *Sphingobacteriaceae* compared to mothers with normal weight. Additionally, we observed that the median bacterial abundance was higher in overweight mothers compared to those with normal weight. Although these differences were not statistically significant overall, stratified analysis at the family level revealed distinct patterns, with overweight mothers showing greater microbial richness in their milk. This finding aligns with previous research by Lundgren et al. [39], who reported a positive association between gestational weight gain and bacterial alpha diversity in HM. In turn, a Mexican study [29] reported a negative correlation trend between the phylum Firmicutes and weight gain during pregnancy, bicipital skinfold, and suprailiac skinfold (*p* < 0.2). These associations may reflect systemic metabolic influences on mammary gland ecology, potentially mediated through hormonal, immunological, or epithelial mechanisms [17,39]. In contrast, the Firmicutes to Bacteroidota ratio displayed a positive trend with weight gain throughout pregnancy. In addition, the authors observed that adiposity concentrated in the upper body, as indicated by the bicipital and subscapular skinfold measurements, showed a significant positive correlation with Bacteroidota and Actinobacteriota when the skinfolds exceeded 1 standard deviation.

Although maternal age did not show a statistically significant effect on relative microbial abundance in our analysis—a finding consistent with previous studies [7,9,40]—a stratified examination revealed age-related trends that were not always aligned with earlier reports. For instance, milk from younger mothers (<35 years) tended to have higher microbial abundance and a distinct taxonomic profile compared to milk from older mothers (>35 years), although these differences were not statistically significant in our small cohort. Similar trends were reported by Li et al. [41], who found the highest bacterial diversity in mothers aged 22–24. Conversely, Ojo-Okunola et al. [42] reported significantly greater Shannon diversity in milk from mothers ≥35 years in a South African cohort. Cortés-Macías et al. [43] also observed higher milk microbiota diversity in mothers in their 30s compared to younger women; however, it is noteworthy that 85% of their cohort was aged 30–40 years. At the family level, our data showed that mothers over 35 had lower overall microbial abundance, with higher levels of *Pseudomonadaceae.* In contrast, milk from mothers under 35 showed increased levels of *Streptococcaceae* and *Staphylococcaceae*, particularly within the 30–35 age range. Interestingly, other studies have reported conflicting genus-level patterns. For example, the genus *Streptococcus* was found in higher abundance in adults (>19 years) [7] and older mothers (>35 years) [43], while *Staphylococcus* abundance negatively correlated with age in mothers under 30 and those aged 30–35 years [43]. It is important to note that our subgroup of mothers over 35 was small (*n* = 3), which may limit the generalizability of these findings. Nevertheless, the observed age-related trends may reflect underlying hormonal and immunological changes across the reproductive lifespan that could influence the composition of the HM microbiota. Overall, our findings support the possibility that maternal age may play a role in shaping HM microbiota, in agreement with prior research.

We also examined the impact of breastfeeding practices—comparing direct breastfeeding to a combination of direct and expressed milk feeding. While overall microbial abundance did not differ significantly between the two groups, specific microbial taxa showed compositional differences. Notably, directly breastfed milk tended to contain a higher median bacterial load than milk obtained via pumping. Our findings are consistent with those of Moossavi et al. [9] in a Canadian cohort, where indirect breastfeeding was independently associated with lower bacterial richness and diversity. The underlying mechanisms behind these observations are not entirely clear, particularly since some of the literature suggests that indirect feeding may introduce environmental taxa into HM. For example, mothers using non-sterile personal breast pumps were found to significantly increase the proportion of milk samples with high bacterial loads [44]. This indicates that pumping can introduce skin- or pump-associated bacteria into the milk. In contrast, direct breastfeeding can promote the transfer of the infant’s oral and skin microbes back into the breast. In our cohort, mothers who used both direct and expressed feeding showed higher relative abundances of *Sphingomonadaceae* and *Sphingobacteriaceae*, and markedly lower levels of *Streptococcaceae* and *Enterobacteriaceae* compared to those who exclusively breastfed directly. Similarly, Moossavi et al. [9] reported that *Enterobacteriaceae* was relatively more abundant in milk from mothers who used indirect feeding methods. Our findings support the idea that pumping, as opposed to direct feeding, may reduce the presence of oral-origin taxa (such as *Streptococcus*) while enriching environmental taxa (such as *Pseudomonadaceae*) in HM.

Among various lifestyle factors related to mothers, dietary habits are most linked to the composition of HM microbiota [18]. Regarding the fact that maternal dietary habits can directly affect the composition of the gastrointestinal microbiome, their diet can also impact HM microbiota diversity through the enteromammary pathway [45]. Contrary to studies demonstrating significant effects of dietary fiber on HM microbial composition [17,18,46,47], our findings revealed no statistically significant differences in microbial composition between high (>24 g/day) and low (<24 g/day) fiber intake groups (*p* = 0.8977). In turn, LeMay-Nedjelski et al. [46] observed that each 1 g increase in fiber from grains was linked to a decrease in the occurrence of Fusobacteria, with an incidence rate ratio (IRR) of 0.86 (95% CI: 0.77 to 0.97). It was also reported [18] that higher fiber intake was associated with increased HM microbial diversity and richness. These results may have specific clinical implications, as a study by Dzidic et al. [48] linked reduced HM microbial richness in the first month of life with a higher risk of allergy in infants. Discrepancies between our results and those of earlier studies may be attributed to differences in study design, sample size, population characteristics, or duration of dietary assessment.

Despite the absence of an observable effect of dietary fiber in our cohort, we identified several significant associations between nutrient intake and HM microbial taxa. Specifically, the phylum Firmicutes was positively correlated with animal protein, carbohydrates, vitamin A (retinol), beta-carotene, starch, and MUFAs. This aligns with evidence that maternal macronutrient intake can act as a selective force on the milk microbiota [17,18]. In a Spanish study [17], the *Streptococcus* genus was directly associated with a higher intake of eicosapentaenoic acid (r = 0.19, *p* = 0.040) and docosapentaenoic acid (r = 0.19, *p* = 0.044) as well as higher total protein intake (r = 0.22, *p* = 0.018), selenium (r = 0.26, *p* = 0.005), and zinc (r = 0.18, *p* = 0.049), whereas the *Staphylococcus* genus was associated with higher intake of carbohydrates (r = 0.19, *p* = 0.038) and with lower total protein intake (r = −0.21; *p* = 0.026). Different results were obtained in one of the recent studies by Marsh et al. [49] who evaluated whether vegan, vegetarian, and omnivore diets impact the HM microbiota. Interestingly, they found that HM microbial diversity was not associated with diet type. However, maternal dietary fatty acid intake had an impact on the HM microbiota.

In addition to quantitative nutrient intake, the frequency of specific food group consumption (Table 2) revealed multiple associations with HM microbiota composition. Our results are consistent with several studies; for instance, Padilha et al. [18] conducted a cross-sectional study with 94 healthy lactating women and found that maternal diet during pregnancy and lactation modulated HM microbiota diversity and composition. Specifically, higher vitamin C intake was associated with increased microbial diversity and a greater abundance of *Staphylococcus* genera (*p* = 0.01). In our study, the association between plant oils, which are rich in unsaturated fats and potentially antioxidants like vitamin E [50], and *Staphylococcaceae* may reflect a similar nutrient-driven effect, although direct nutrient measurements were not conducted. Similarly, some studies reported [17] that maternal diets high in plant protein, fiber, and carbohydrates were associated with greater microbial richness (*p* < 0.0001) and diversity (*p* < 0.0003) in HM compared to diets high in animal protein and lipids. Our finding that wholegrain consumption, a source of fiber and complex carbohydrates, was linked to *Sphingobacteriaceae, Xanthomonadaceae,* and *Enterobacteriaceae* could support this trend. However, greater overall bacterial abundance was observed in association with a higher-fat diet, including foods such as butter, fish, and plant oils, which corresponded with increased levels of *Streptococcaceae*, *Staphylococcaceae*, *Bacillaceae*, and *Sphingobacteriaceae*. Additionally, some bacterial families identified in our study, such as *Sphingomonadaceae* and *Sphingobacteriaceae*, are less commonly reported in maternal diet alterations in the HM microbiota literature.

Given the pilot nature of this study, the findings should be interpreted with caution. They provide a foundation for further investigation into how dietary patterns and body composition influence human milk microbiota. Nonetheless, given the limited research on HM composition in the Polish population [51], this work represents one of the first such analyses conducted in Poland. In addition to the small sample size, there are additional limitations. The microbial profiles were assessed by 16S rRNA gene amplicon sequencing, a culture-independent method that is unable to differentiate between live and dead bacteria or detect cell-free DNA [52]. Future studies confirming our results should try to isolate species identified using culturing techniques. This study provides a valuable basis for further research. We plan to expand the sample size and extend the analysis to include other microbial groups, such as fungi, to gain a more comprehensive understanding of the HM microbiome.

## 4. Materials and Methods

### 4.1. Study Design

Mothers were recruited from the Department of Obstetrics and Gynaecology in the Specialistic Holy Family Hospital in Warsaw during their stay in the maternity ward, in cooperation with the midwifery service. Initially, we recruited 22 women; however, seven did not meet the inclusion criteria (age ≥ 18 years, no chronic diseases, singleton pregnancy, no smoking during or after pregnancy, term pregnancy, sufficient milk supply, no use of antibiotics and probiotics in the last 3 months, and exclusive breastfeeding). Therefore, the final analysis involved 15 mothers. Consequently, a total of 15 mothers were involved in the final analysis. This sample size was chosen based on the exploratory nature of this pilot study, aiming to gather preliminary data on the relationship between maternal dietary habits, body composition, and the microbial composition of human milk. All women received written instructions for standardized milk collection and nutritional questionnaires.

The study session with a clinical dietetic (A.B.-J.) took place between the third and fourth week postpartum, during a maternal visit to the Medical University of Warsaw. During these meetings, mothers were weighed and measured (height), and the body composition analysis was performed. Additionally, women were asked about sociodemographic and perinatal data, and a nutritional questionnaire was verified.

### 4.2. Maternal Anthropometric Measurements and Body Composition Analysis

Maternal body weight and height were measured using a Seca 799 electronic column scale (±0.1 kg/cm, Seca, Hamburg, Germany). An innovative aspect of this pilot study is the assessment of maternal nutritional status based on body composition analysis rather than the commonly used BMI metric, allowing for a more nuanced understanding of how maternal health influences human milk microbiota. Body composition analysis was based on bioelectrical bioimpedance (BIA) and performed using a Maltron BioScan 920-II instrument (Maltron Bioscan, Rayleigh, UK) under a validated protocol [53]. Total body electrical impedance alternated between four frequencies, namely 5, 50, 100, and 200 kHz, and the resistance and reactance of the vector components were measured simultaneously. The following parameters were obtained: fat mass (kg, %), fat-free mass (kg, %), total body water (L, %), extra- and intracellular water (L, %), muscles (kg), proteins (kg), and minerals (kg). Furthermore, resting energy expenditure for each mother was calculated.

### 4.3. Maternal Nutritional Data

The nutritional questionnaire was divided into two parts. The first one was based on a semi-structured food frequency questionnaire (FFQ) for habitual intake, and the second one was a 3-day dietary record of current intake. A semi-structured FFQ was developed by the WHO guidelines [54] and used to assess the consumption of a selected group of food products for the 3 months before the study, which involved the last two months of pregnancy and one month of lactation. Considering current intake, for the calculation of energy and nutrient consumption, the Polish reference method was used (Dieta 6.0 nutritional software, National Food and Nutrition Institute, Warsaw, Poland). The primary goal of the Dieta 6.0 software is to facilitate the assessment of the energy and nutritional content of diets, along with the evaluation of food quantities and consumption patterns. This is achieved by contrasting the computed nutritional values with established guidelines and adopting a fresh perspective on the adequacy of nutrient intake evaluation, utilizing probability and cut-off methods [55].

### 4.4. Collection of Human Milk Samples

Samples of mature HM were collected between 4–6 weeks postpartum, one day before the mother visited the Laboratory of Human Milk and Lactation Research, on the third day of the 3-day dietary record. Women were asked to fully express milk in the morning (7:00–9:00 a.m.) and immediately freeze it and store it at −20 °C. Before sample collection, women cleaned the breast with an iodine swab to reduce bacteria residing on the maternal skin. Milk was collected manually in a sterile milk container. After delivering samples to the laboratory (the day after the collection), samples were stored for further analysis at −80 °C.

### 4.5. DNA Extraction

Total genomic DNA from milk samples was isolated using the Environmental DNA/RNA Extraction Kit (Eurx, Nottingham, UK) following the manufacturer’s instructions with minor modifications. The milk samples were centrifuged at 14,000 rpm for 5 min, and the supernatant was discarded. The bacterial pellet was resuspended in the provided buffer and mechanically disrupted by pulverization using glass beads in a Tissue Lyser apparatus (Qiagen, Singapore). For cell lysis, the lysozyme solution was supplemented with Mutanolysin (1 U/mL) and Lysostaphin (20 µg/mL) to enhance Gram-positive bacterial lysis. A blank isolation control was included to monitor potential contamination from environmental bacterial DNA. The isolated DNA was quantified by fluorimetry using a Qubit 2.0 Flurometer with High Sensitivity Picogreen reagents (Thermo, Waltham, MA, USA).

### 4.6. 16S rRNA Amplicon Sequencing

Amplification of the conserved bacterial 16S rRNA gene fragment covering V3 and V4 regions was carried out in triplicate using gene-specific primers: 16S_V3-F and 16S_V4-R positions 341–357F and 785–805R, respectively, corresponding to the *Escherichia coli* 16S rRNA gene reference sequence [56]. The resulting amplicons of the size ca. 450 bp were verified by 1% agarose gel electrophoresis and purified with Ampure XP magnetic beads (Beckman, Pasadena, CA, USA). Amplicon libraries were pooled in equimolar ratios and indexed according to the Nextera indexing strategy by PCR protocol (Illumina, San Diego, CA, USA). The indexing strategy enabled pooling of amplicons for sequencing and subsequent extraction of sample-specific reads from the sequencing data. The 16S amplicons were sequenced on an Illumina MiSeq platform at the DNA Sequencing and Oligonucleotide Synthesis Laboratory (IBB PAS) using a 600-cycles v3 chemistry kit (Illumina, San Diego, CA, USA) in paired-end mode. Blank isolation samples, along with PCR-negative and -positive controls, were sequenced alongside experimental samples on the Illumina platform. Sequence reads were quality-checked using FastQC v.0.12.1 toolkit [57] and processed through the Qiime2 v.2021.11 pipeline [23]. Briefly, paired-end reads were merged and denoised using DADA2 v.1.26.0.

### 4.7. Data Analysis and Availability

Sequences were taxonomically classified using a pre-trained Bayesian Naïve classifier based on the SILVA database (“silva-138-99-nb-classifier.qza a”, https://docs.qiime2.org/2021.11 /data-resources/, accessed on 30 September 2022).

Downstream analysis was performed in the R environment. The Decontam v.1.12 package was used for data curation based on results obtained from the negative control (https://benjjneb.github.io/decontam/, accessed on 30 September 2022).

Raw data (FASTQ files) were deposited in the NCBI under BioProject PRJNA888867 (BioSamples SAMN31222700-SAMN31222714). Illumina SRA reads are available under the accession numbers: SRR21902580–SRR1902594.

### 4.8. Statistical Analysis

All statistical analyses and data visualizations were conducted using GraphPad Prism version 9.0 (GraphPad Software, San Diego, CA, USA) and Microsoft Excel with the Analyze-it^®^ add-in (Analyse-it Software, Ltd., Leeds, UK) for advanced statistical procedures. Non-parametric statistical tests were employed throughout the study to account for the non-normal distribution of microbial abundance data.

Microbial diversity and between-group comparisons of bacterial abundances were assessed using the Mann–Whitney U test for two independent groups (e.g., age categories, BMI groups, delivery modes, breastfeeding types, and fiber intake levels), followed by false-discovery rate (FDR) correction using the Benjamini, Krieger, and Yekutieli method, with a desired FDR set at 5%.

To evaluate relationships between nutrient intake, body composition parameters, and microbial taxa, Kendall tau correlation coefficients were calculated. This non-parametric rank-based method was chosen due to the small sample size and the non-normal distribution of the data. Analyses were performed using the Analyze-it^®^ statistical add-in for Excel. For each pairwise comparison, the Kendall tau correlation coefficient (τ) and the associated *p*-value are reported. Statistical significance was set at *p* < 0.05.

Dietary habits of 15 women were categorized based on consumption frequency of specific food groups: 1 (very often), 2 (often), 3 (once per week), 4 (a few times per month), and 5 (once per month or rare). The data were further divided into two categories (1,2 vs. 3,4,5 or 1,2,3 vs. 4,5). Microbiota abundance between these two groups was compared using the Mann–Whitney U test.

## 5. Conclusions

This pilot study highlights the significance of maternal dietary habits and body composition in shaping the microbiota of human milk. Our findings revealed preliminary correlations between specific nutrient intakes and the abundance of microbial taxa, which may have important implications for infant health and development. By utilizing body composition analysis as a measure of maternal nutritional status rather than the commonly employed BMI metric, we provide a more nuanced understanding of how maternal health influences human milk microbiota.

While the small sample size limits the generalizability of our results, the insights gained from this pilot study lay the groundwork for future research. Further investigations with larger cohorts are necessary to validate our findings and explore the underlying mechanisms that govern the relationship between maternal diet, body composition, and the human milk microbiota. Ultimately, this research paves the way for a deeper understanding of maternal health and its impact on offspring, contributing to the development of targeted dietary recommendations for breastfeeding mothers.

## Figures and Tables

**Table 1:** The characteristics of mothers and infants included in the study (*n* = 15).

Parameters	Mean ± SD	Median (IQR)
Maternal characteristics		
Age (years)	32.1 ± 5.0	31 (29–33)
Pre-pregnancy BMI (kg/m^2^)	21.2 ± 2.7	20.8 (19.1–22.6)
Weight gain during pregnancy (kg)	14.0 ± 2.6	14 (12–16)
Actual BMI (kg/ m^2^)	22.4 ± 2.8	22.3 (19.8–23.8)
Fat-free mass (kg)	44.0 ± 3.2	44.6 (42.4–45.6)
Fat-free mass (%)	71.8 ± 6	70.7 (67.8–75.7)
Fat mass (kg)	17.9 ± 6.1	18 (13.4–21.7)
Fat mass (%)	28.2 ± 6.0	29.3 (24.3–32.2)
Total body water (L)	30.7 ± 2.7	30.5 (28.9–32)
Total body water (%)	49.9 ± 3.9	49.5 (47.1–51.5)
Extracellular body water (L)	14.2 ± 1.5	14.5 (12.9–15.3)
Extracellular body water (%)	46.3 ± 1.8	47 (45.4–47.6)
Intracellular body water (L)	16.5 ± 1.5	16.3 (15.9–16.9)
Intracellular body water (%)	53.7 ± 1.8	52.9 (52.3–54.6)
Protein (kg)	9.5 ± 0.8	9.7 (8.7–10.1)
Minerals (kg)	3.9 ± 0.3	4 (3.5–4.1)
Muscle mass (kg)	18.9 ± 2.0	19.6 (18.4–19.9)
Resting metabolic rate (kcal)	1509.5 ± 49.7	1513 (1481.5–1532)
Infant characteristics		
Birth weight (g)	3553.7 ± 401.3	3550 (3185–3827.5)
Birth height (cm)	55.3 ± 1	55 (54.5–56)
Number of day feedings	8.8 ± 3.5	8 (7–9.5)
Number of night feedings	1.9 ± 0.6	2 (2–2)

**Table 2:** The characteristics of maternal dietary intake.

	Mean ± SD	Median (IQR)
Macronutrients		
Total protein (g)	81.91 ± 24.2	74.39 (62.91–102.5)
Animal protein (g)	50.6 ± 17.26	44.89 (37.44–64.21)
Plant protein (g)	30.75 ± 10.36	27.22 (23.14–32.6)
Total fat (g)	68.7 ± 17.91	63.92 (57.91–82.95)
Cholesterol (mg)	289 ± 123.5	289.2 (208–343.8)
Saturated fatty acids (SFA) (g)	25.91 ± 8.34	26.05 (19.06–31.5)
Monounsaturated fatty acids (MUFA) (g)	26.28 ± 8.467	24.89 (18.69–34.05)
Polyunsaturated fatty acids (PUFA) (g)	11.61 ± 5.284	10.7 (8.94–13.15)
Long-chain polyunsaturated fatty acids (g)	0.3385 ± 0.6199	0.073 (0.03–0.2773)
Total carbohydrates (g)	260.5 ± 66.3	245.9 (216.4–298.6)
Sucrose (g)	41.57 ± 20.11	39.56 (26.71–55.49)
Lactose (g)	10.99 ± 9.232	10.48 (2.54–17.98)
Starch (g)	136.4 ± 36.55	126.6 (111.5–173.8)
Fiber (g)	22.28 ± 6.821	21.21 (16.77–25.13)
Energy from protein (%)	17.06 ± 2.726	17.3 (15.35–19.23)
Energy from fat (%)	31.54 ± 4.606	31.11 (27.81–32.57)
Energy from carbohydrates (%)	51.09 ± 5.751	50.73 (47.68–56.84)
Micronutrients		
Sodium (mg)	2862 ± 1043	2892 (1962–3548)
Potassium (mg)	3202 ± 893.5	3092 (2485–3573)
Calcium (mg)	818 ± 354.3	723.1 (590.4–1030)
Phosphorus (mg)	1402 ± 386.8	1230 (1182–1620)
Magnesium (mg)	347.3 ± 129.1	301.1 (262.4–391.6)
Iron (mg)	16.06 ± 14.79	11.55 (10.04–14.55)
Zinc (mg)	11.95 ± 6.289	10.95 (7.276–12.79)
Manganese (mg)	5.287 ± 2.204	4.61 (3.54–6.53)
Iodine (μg)	118 ± 47.3	107.4 (75.31–154.2)
Beta-carotene (μg)	4542 ± 2402	3786 (3345–5568)
Folic acid (μg)	380.7 ± 206.8	308.7 (262.6–417.8)
Retinol (μg)	361.3 ± 130.9	359 (285.2–407.8)
Vitamin A (μg)	1110 ± 484.9	1007 (780.4–1284)
Vitamin B_6_ (mg)	2.167 ± 0.9334	1.729 (1.459–2.44)
Vitamin B_12_ (μg)	3.758 ± 2.187	2.915 (2.44–4.8)
Vitamin C (mg)	137.3 ± 100.9	123.2 (87.63–151)
Vitamin D (μg)	4.266 ± 3.71	2.549 (1.63–5.618)
Vitamin E (mg)	11.46 ± 4.27	10.87 (7.54–14.75)
Thiamine (mg)	1.401 ± 0.6372	1.147 (0.91–1.56)
Riboflavin (mg)	1.886 ± 0.6132	1.64 (1.53–2.038)
Niacin (mg)	17.98 ± 7.996	15.09 (10.52–23.5)

**Table 3:** Associations between significant bacterial taxa in human milk and dietary intake frequency categories (1, 2, and 3). The table displays microbial composition based on food group consumption frequency.

Food Group	Bacteria Family	*p*-Value
Animal milk	*Streptococcaceae*	0.072
	*Sphingomonadaceae*	0.005 *
Plant oils	*Streptococcaceae*	0.088
	*Staphylococcaceae*	0.002 *
Oily fishes	*Bacillaceae*	0.069
Lean fishes	*Bacillaceae*	0.052
	*Sphingobacteriaceae*	0.131
Butter	*Bacillaceae*	0.103
	*Staphylococcaceae*	0.073
Wholegrain	*Sphingobacteriaceae*	0.111
	*Xanthomonadaceae*	0.059
	*Enterobacteriaceae*	0.018 *
Buckweats	*Sphingobacteriaceae*	0.035 *
peantuts	*Xanthomonadaceae*	0.027 *
	*Sphingobacteriaceae*	0.029 *
Poultry	*Enterococcaceae*	0.038 *

* *p* < 0.05.

## Data Availability

The data presented in this study are available on request from the corresponding author due to privacy restrictions.
